# 供者CD19 CAR-T细胞治疗急性B淋巴细胞白血病移植后复发九例临床观察

**DOI:** 10.3760/cma.j.issn.0253-2727.2021.05.006

**Published:** 2021-05

**Authors:** 润芝 马, 祎 何, 栋林 杨, 嘉璘 魏, 爱明 庞, 尔烈 姜, 建祥 王, 明哲 韩, 荣莉 张, 四洲 冯

**Affiliations:** 中国医学科学院血液病医院（中国医学科学院血液学研究所），实验血液学国家重点实验室，国家血液系统疾病临床医学研究中心，天津 300020 State Key Laboratory of Experimental Hematology, National Clinical Research Center for Blood Diseases, Institute of Hematology & Blood Diseases Hospital, Chinese Academy of Medical Sciences & Peking Union Medical College, Tianjin 300020, China

**Keywords:** 嵌合抗原受体, 异基因造血干细胞移植, 急性淋巴细胞白血病, CD19, 复发, CAR-T cell, B cell acute lymphoblastic leukemia, Allogeneic hematopoietic stem cell transplantation, CD19, Relapse

## Abstract

**目的:**

观察供者抗CD19嵌合抗原受体T细胞（CAR-T）（HI19α-4-1BB-ζ CAR-T）治疗急性B淋巴细胞白血病（B-ALL）异基因造血干细胞移植（allo-HSCT）后复发患者的疗效及安全性。

**方法:**

对2017年7月至2020年5月期间9例allo-HSCT后复发B-ALL患者应用供者抗CD19 CAR-T细胞治疗，FCA方案（氟达拉滨+环磷酰胺+阿糖胞苷）预处理后回输供者CD3^+^T淋巴细胞，其中CAR-T细胞中位数1.79（0.86～3.53）×10^6^/kg，观察疗效和不良反应。

**结果:**

①输注CAR-T细胞后28～42 d，9例患者均获得MRD阴性的完全缓解。②所有患者发生细胞因子释放综合征（CRS），其中3级2例、2级4例、1级3例；4例患者出现免疫效应细胞相关的神经毒性（ICANS），2级1例、1级3例；1例患者发生急性Ⅳ度移植物抗宿主病（GVHD），上述不良反应经治疗均控制。③4例患者再次复发，中位复发时间为CAR-T细胞治疗后8.6（4.6～19.3）个月，2例化疗后病情进展死亡，1例接受二次移植14个月后复发死亡，1例接受CD22 CAR-T细胞治疗后完全缓解，现6例患者无病存活，植入分析为完全供者嵌合体，中位无白血病生存（LFS）期18.1个月，预期1年、2年LFS率分别为63.5％、50.8％。④中位随访25.1（6.9～36.7）个月，预期2年、2.5年总生存（OS）率分别为87.5％、52.5％。

**结论:**

供者抗CD19 CAR-T细胞治疗allo-HSCT后复发的B-ALL的缓解率高，不良反应可耐受，半数患者可无病生存2年以上，长期疗效有待进一步观察。

## Abstract (en)

**Objective:**

To investigate the long term efficacy and side effects of a donor-derived CD19 chimeric antigen receptor（CAR）T-cell（HI19α-4-1BB-ζ CAR-T）therapy in the treatment of patients with relapsed B-cell acute lymphoblastic leukemia（B-ALL）after allogeneic hematopoietic stem cell transplantation（allo-HSCT）.

**Methods:**

A total of 9 subjects with relapsed B-ALL post allo-HSCT received donor-derived CD19 CAR-T therapy from July 2017 to May 2020. All subjects were infused with donor CD3-positive T cells after lymphodepletion chemotherapy, and a median dose of CAR-T cells was 1.79（range, 0.86–3.53）×10^6^/kg.

**Results:**

①All subjects achieved complete remission and MRD-negative at 28–42 d post CAR-T cells infusion. ②Cytokine releasing syndrome（CRS）occurrd in all subjects and was grade 3 in 2, grade 2 in 4, grade 1 in 3 cases respectively. Four subjects developed immune effector cell-associated neurotoxicity syndrome（ICANS）, which was grade 2 in 1, grade 1 in 3. One subject developed grade IV acute graft-versus-host disease（GVHD）, and side effects were all controllable. ③Four subjects relapsed at a median period of 8.6（4.6–19.3）months, 2 subjects died of disease progression after receiving chemotherapy and another one also died of disease progression 14 months after a second transplant, only 1 subject achieved complete remission after CD22 CAR-T cell therapy. Until last follow-up date, 6 subjects were leukemia-free and achieved complete donor chimerism. The estimated 1-year and 2-year leukemiafree survival（LFS）rate was 63.5％ and 50.8％, with a median LFS of 18.1 months. ④After a median follow-up of 25.1（range, 6.9–36.7）months, the estimated 2-year and 2.5-year OS rate were 87.5％ and 52.5％, respectively.

**Conclusion:**

The donor-derived CD19 CAR-T cell therapy obtain a high remission rate in relapsed B-ALL patients post allo-HSCT with tolerable side effects, half subjects survived more than 2 years without disease recurrence, though long-term efficacy requires further observation.

20％～40％的急性淋巴细胞白血病（ALL）患者异基因造血干细胞移植（allo-HSCT）后复发，复发后患者的预后极差，中位总生存（OS）期约为5.5个月[1]。靶向CD19嵌合抗原受体T细胞（CAR-T细胞）治疗复发难治ALL患者完全缓解率达83％，中位OS期12.9个月[2]，若桥接移植可延长生存获益[3]。CD19 CAR-T细胞（HI19α-4-1BB-ζ CART，CNCT19）免疫治疗在难治复发B-ALL患者疗效显著[4]。然而，CD19 CAR-T细胞治疗allo-HSCT后复发的ALL患者报道不多，如何延长供者CAR-T细胞的体内存留时间、细胞因子释放综合征（CRS）和急性移植物抗宿主病（aGVHD）等并发症的处理，以及如何治疗CAR-T细胞治疗后再复发，仍是目前亟待解决的问题。我们采用新采集供者细胞构建CD19 CAR-T细胞，用于治疗B-ALL患者移植后复发并取得了一定疗效，现报道如下。

## 病例与方法

1. 病例资料：回顾性分析2017年7月至2020年5月在我院采用供者CD19 CAR-T细胞治疗的allo-HSCT后复发B-ALL患者资料。9例复发患者的白血病细胞流式细胞术（FCM）检测均表达CD19。移植后复发的定义：①血液学复发：外周血中出现幼稚细胞或骨髓幼稚细胞（blast）比例超过5％。②分子学生物学复发微小残留病（MRD）阳性：骨髓blasts≤5％、幼稚淋巴细胞可被多色FCM检测到（>0.01％）或实时定量PCR（qPCR）检测特异性基因（如BCR-ABL融合基因）阳性。③骨髓外复发（EMR）：单部位活检证实独立髓外复发或PET-CT证实的多部位骨髓外复发。所有患者均入组临床试验XH-CAR-T-003（中国临床试验注册中心：ChiCTR1900025419），且本研究获得中国医学科学院血液病医院伦理委员会批准。

2. allo-HSCT方案：预处理方案：①全身照射（TBI）+环磷酰胺（Cy）+氟达拉滨（Flu）+阿糖胞苷（Ara-C）：TBI 3.3 Gy/d，−9～−7 d；Cy 40 mg·kg^−1^·d^−1^，−6～−5 d；Flu 30 mg · m^−2^·d^−1^，−4 ～ −2 d；Ara-C 2 g·m^−2^·d^−1^，−4～−2 d。②白消安（Bu）+Cy+依托泊苷（Vp16）：Bu 3.2 mg·kg^−1^·d^−1^，−9～−7 d；Cy 40 mg·kg^−1^·d^−1^，−6～−5 d；Vp16 30 mg/kg，−7 d。本组中8例采用方案①、例8采用方案②。亲缘单倍型移植联合兔抗人胸腺细胞球蛋白（rATG）2.5 mg·kg^−1^·d^−1^×（3～4）d。预处理后回输供者外周血动员的造血干细胞，单个核细胞中位输注量为10.5（7.0～28.5）×10^8^/kg，CD34^+^细胞的中位输注量为2.5（1.4～4.4）×10^6^/kg。同胞全相合移植采用环孢素A（CsA）+短疗程甲氨蝶呤（MTX）预防移植物抗宿主病（GVHD），亲缘单倍型移植采用霉酚酸酯（MMF）+CsA或他克莫司+短疗程MTX预防GVHD。

3. 供者CAR-T细胞的制备：新采集9例移植后复发患者的供者外周血100～200 ml并立即分离T淋巴细胞，由天津合源生物科技有限公司制备CD19 CAR-T细胞。本研究采用的CAR单链可变区（scFv）来源于HI19α。具体为Anti-CD19 scFv与人4-1BB/CD3-ζ共刺激结构域共同组成CAR片段，构建慢病毒载体（pCDH-HI19α-4-1BB/CD3ζ-CAR）、转染供者T细胞并进行体外扩增。制备的供者CD19 CAR-T细胞复检确认无细菌、真菌、支原体、衣原体和内毒素污染后，冻存于−80 °C备用。

4. CAR-T细胞治疗方案：多数患者在CD19 CAR-T细胞治疗前接受FCA方案（Flu 30 mg·m^−2^·d^−1^，−4～−2 d；Cy 300～350 mg·m^−2^·d^−1^，−4、−2 d；Ara-C 100 mg·m^−2^·d^−1^，−4～−1 d）清淋巴细胞预处理；例8采用FC（Flu 30 mg·m^−2^·d^−1^，−4～−1 d；Cy 500 mg·m^−2^·d^−1^，−4～−3 d）；例9系肿瘤负荷高的年轻患者，FA方案（Flu 30 mg·m^−2^·d^−1^，−4～−2 d；Ara-C 250 mg·m^−2^·d^−1^，−4～−2 d）预处理后2～3 d回输供者来源CAR-T细胞，Anti-CD19/4-1BB CAR-T细胞中位数为1.79（0.86～3.73）×10^6^/kg，回输后不进行GVHD预防，FCM检测CAR-T细胞的扩增和存留。

5. 不良反应和疗效评估：不良反应主要包括CRS，CRS分级参照Lee等[5]的分级标准进行评价，免疫效应细胞相关的神经毒性（ICANS）及其他不良反应参照NCI-CTCAE v5.0（https://ctep.cancer.gov/）进行评估，观察周期为24个月。GVHD分级采用美国西雅图标准，糖皮质激素作为GVHD一线治疗方案。患者回输CAR-T细胞后28～42 d进行骨髓检查确定缓解状态，之后每1～3个月评估：骨髓细胞形态学、FCM检测异常免疫表型残留细胞、qPCR检测融合基因、短串联重复序列多态性（STR）检测供者植入状态。完全供者嵌合体定义为STR检测供者细胞比例≥97％。例6因发生EMR，回输后每2个月接受PET-CT评估复发灶变化。疗效评估参考NCCN指南，完全缓解定义为形态学缓解（骨髓blasts<5％）且MRD阴性。复发包括血液学复发或MRD转阳性。

6. 随访和统计学处理：所有患者在CAR-T细胞回输后开始进行随访，OS期定义为CAR-T细胞输注当日至死亡或随访终点，无白血病生存（LFS）期为治疗后达完全缓解（CR）或形态学完全缓解而血细胞计数未完全恢复（CRi）至复发、死亡或随访终点，随访终点为2020年11月20日。采用Kaplan-Meier绘制生存曲线，使用SPSS 25.0软件进行统计学描述。

## 结果

一、病例资料

本研究共入组9例移植后复发的B-ALL患者，其中男3例，女6例，中位年龄44（16～60）岁，其中同胞全相合（MSD）移植6例、亲缘单倍型（Haplo）移植3例。移植前预后危险度评估高危患者7例，复发情况：3例为血液学复发，5例患者骨髓MRD阳性，1例患者骨髓MRD阳性伴EMR。复发后至CAR-T细胞治疗的中位时间为204（6～940）d。CAR-T细胞治疗之前接受化疗1例、供者淋巴细胞输注（DLI）1例、DLI联合化疗2例、DLI联合酪氨酸激酶抑制剂（TKI）2例，DLI回输供者CD3^+^细胞的中位数为5.05（3.77～7.65）×10^7^/kg。CAR-T细胞回输前6例患者的骨髓原始细胞比例为0.01％～5％、3例达5％～50％。CD19 CAR中位转染效率为49.2％（25.0％～70.6％），输注供者CD19 CAR-T细胞的中位数为1.79（0.86～3.53）×10^6^/kg。具体资料见表1～2。

**表1:** 供者CD19 CAR-T细胞治疗9例移植后复发B-ALL患者异基因造血干细胞移植（allo-HSCT）基线资料

例号	性别	年龄（岁）	诊断	危险度和遗传学异常	移植供者类型	复发类型/骨髓肿瘤负荷	移植后复发治疗	复发至CAR-T间隔（d）
1	女	22	B-ALL-CR_2_	高危，TEL/AML1	MSD	MRD阳性/2％	化疗+DLI	204
2	女	44	Ph^+^ALL-CR_1_	高危，BCR-ABL	单倍型	MRD阳性、BCR-ABLP190（+）/0.05％	DLI+伊马替尼	355
3	男	60	Ph^+^ALL-CR_2_	高危，BCR-ABL	MSD	血液学复发/67％	DLI+达沙替尼	834
4	男	47	B-ALL-CR_1_	高危	MSD	血液学复发/73％	化疗+DLI	940
5	女	50	B-ALL-CR_1_	高危，IGK重排、TCRγ重排	MSD	血液学复发/39.5％	无	29
6	女	46	B-ALL-CR_1_	标危，FLT3/ITD	MSD	MRD阳性、EMR/0.05％	DLI	227
7	女	26	B-ALL-CR_1_	标危，无	MSD	血液学复发/26％	化疗	66
8	女	40	B-ALL-CR_1_	高危，DNMT3A、WHSC1、NOTCH1	单倍型	MRD阳性/0.01％	无	53
9	男	16	B-ALL-CR_1_	高危，亚二倍体、复杂核型	单倍型	血液学复发/15％	无	6

注：CAR-T细胞：嵌合抗原受体T细胞；ALL：急性淋巴细胞白血病；CR_1_：第一次完全缓解；CR_2_：第二次完全缓解；MSD：同胞相合供者；MRD：微小残留病，MRD阳性定义为流式细胞术检测异常白血病细胞>0.01％或Ph^+^ALL患者BCL/ABL融合基因转阳；EMR：骨髓外复发，包括PET-CT显示的锁骨上、颈部淋巴结（SUV_max_＝6.1），以及心包（SUV_max_＝9.5）和腹膜（SUV_max_＝11.7）；DLI：供者淋巴细胞输注

**表2:** 供者CD19 CAR-T细胞治疗9例移植后复发B-ALL患者疗效

例号	CAR-T输注量（×10^6^/kg）	CAR-T治疗前状态	CAR-T治疗前骨髓肿瘤负荷（％）	输注1个月疗效	CAR-T后是否复发	预后		
						LFS时间（月）	OS时间（月）	转归
1	1.80	MRD阳性	2.51	CR，STR99.32％	是	18.1	25.1	死亡
2	1.30	MRD阳性	0.15	CR，STR99.45％	否	35.7	36.7	无病生存
3	1.25	血液学复发	17.50	CR，STR98.77％	否	34.6	35.6	无病生存
4	1.79	MRD阳性	0.07	CR，STR99.97％	是	7.4	25.6	死亡^a^
5	2.50	血液学复发	39.50	CR，STR99.92％	否	28.6	29.1	无病生存
6	3.53	MRD阳性、EMR	0.05	CR，STR99.35％	是	7.7	14.8	死亡
7	2.46	MRD阳性	0.69	CR，STR99.94％	否	23.3	24.2	无病生存
8	1.42	MRD阳性	6.24	CR，STR99.98％	是	3.7	15.7	复发后再缓解^b^
9^c^	0.86	血液学复发	15.00	CR，STR99.80％	否	6.3	6.9	无病生存

注：CAR-T细胞：嵌合抗原受体T细胞；B-ALL：急性B淋巴细胞白血病；LFS：无白血病生存；OS：总生存；CR：完全缓解；STR：短串联重复序列多态性。^a^例4 CAR-T治疗复发后行二次移植14个月后白血病再次复发并进展死亡；^b^例8 CD19 CAR-T治疗复发后行CD22 CAR-T细胞治疗后再缓解、无病生存；^c^例9回输后6 d发生移植物抗宿主病，累及肠道、肝脏和皮肤

二、不良反应

所有患者发生Ⅲ～Ⅳ级血液学不良反应，8例发生肝功能异常（转氨酶升高），1例发生肾功能异常（血肌酐升高），4例患者有凝血功能异常，经对症支持治疗均恢复正常。

本研究组9例患者均发生CRS，其中3级2例、2级4例、1级3例，CRS在回输1周内出现，中位持续时间6（3～10）d。所有患者回输CAR-T细胞后出现发热、疲乏，中位持续5（3～10）d。5例患者（例1、3、4、7、8）出现间歇性低血压，3级2例、1级3例，静脉应用去甲肾上腺素和托珠单抗（8 mg/kg）后恢复正常。例8回输后21 d出现迟发型CRS，表现为浆膜腔积液（腹水）、肝功能异常（转氨酶升高、胆红素升高）、凝血功能异常，伴CAR-T细胞比例和血清IL-6、IL-2R上升，予地塞米松、保肝、补充血浆和纤维蛋白原等处理后缓解。例6输注CAR-T细胞后出现严重CRS（3级），+6 d出现呼吸困难、低氧血症、腹痛、低体温伴多浆膜腔积液，血清IL-2R>7500 U/ml、IL-6>1000 ng/L，予托珠单抗单次8 mg/kg无效，+8 d出现肠梗阻及心包、胸腔积液，予胸腔积液穿刺引流、甲泼尼龙（1.5 mg·kg^−1^·d^−1^×3 d）等处理，症状逐渐缓解，+18 d出现神经毒性，应用地塞米松2 d后逆转。

4例患者出现ICANS，1级3例、2级1例，对症支持或应用地塞米松后神经症状缓解。仅例9发生Ⅳ度急性GVHD，累及肠道、肝脏和皮肤，给予糖皮质激素并联合环孢素A、巴利昔单抗治疗后好转，其他患者无新发GVHD（表3）。

**表3:** 供者CD19 CAR-T细胞治疗9例移植后复发B-ALL患者的不良反应

例号	CRS			GVHD	ICANS	不良反应及分级						
	分级	托珠单抗	持续时间（d）			发热	疲乏	低血压	低氧血症	心功能不全	ALT或AST升高	凝血功能异常
1	2	是	3	无	1级	3	1	3	0	0	2	0
2	1	否	10	无	无	1	1	0	0	0	1	0
3	2	是	8	无	1级	2	2	3	2	2^b^	1	3
4	2	是	4	无	无	2	1	1	0	0	1	0
5	1	否	5	无	无	2	1	0	0	0	0	0
6	3	是	7	无	2级	2	2	0	1	3^b^	1	3
7	2	是	6	无	无	2	1	1	0	0	2	0
8	1	是	5	无	1级	3	1	1	0	0	0	0
	3^a^	否	10	无	无	1	2	0	0	0	4	2
9	1	否	7	Ⅳ度	无	2	1	0	0	0	4	2

注：CAR-T细胞：嵌合抗原受体T细胞；B-ALL：急性B淋巴细胞白血病；CRS：细胞因子释放综合征；CVHD：移植物抗宿主病；ICANS：免疫效应细胞相关神经毒性；ALT：谷丙转氨酶；AST：谷草转氨酶。^a^例8于CAR-T细胞输注后3 d、21 d 发生2次CRS过程；^b^例3、例6心功能不全以心包积液为主要表现；^c^凝血功能异常包括活化的部分凝血酶原时间（APTT）延长、凝血酶时间（TT）延长和纤维蛋白原降低

三、疗效评估和生存情况

所有患者回输CAR-T细胞28～42 d后均达到骨髓MRD阴性和完全供者嵌合，CR率100.0％（9/9），且FCM检测外周血CD19^+^B淋巴细胞维持在极低水平或低于检测阈值。例6 CAR-T治疗前PET-CT显示锁骨上、颈部淋巴结（SUV_max_＝6.1）、心包（SUV_max_＝9.5）和腹膜（SUV_max_＝11.7）均存在EMR灶，CAR-T治疗后2个月PET-CT显示EMR灶基本消失（图1）。

**图1:**
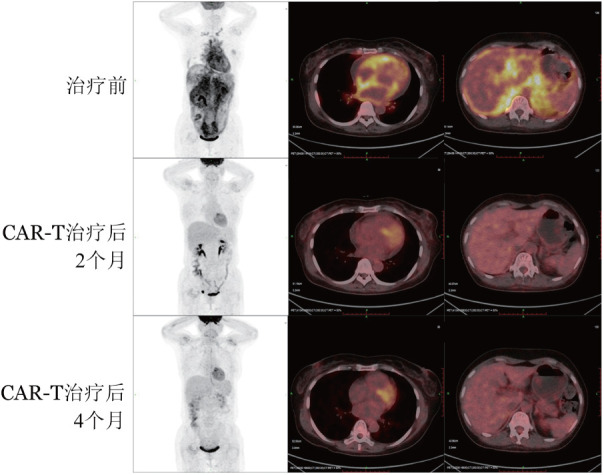
PET-CT示患者（例6）CD19 CAR-T细胞治疗后骨髓外复发病灶缓解 CAR-T细胞：嵌合抗原受体T细胞

中位随访25.1（6.9～36.7）个月，4例患者（例1、4、6、8）分别在CAR-T细胞输注后19.3、8.4、8.8和4.6个月出现再次复发且为CD19阴性，中位复发时间8.6个月。再次复发后，2例患者（例1、6）接受化疗后白血病进展死亡，例4化疗后再次CR行亲缘单倍型移植，二次移植后14个月后白血病进展死亡，仅例8接受CD22 CAR-T细胞治疗后无病生存。随访结束时9例患者中6例患者无病生存，植入分析均是完全供者嵌合体。中位无白血病生存（LFS）期18.1（3.7～35.7）个月，预期1年、2年LFS率分别为63.5％、50.8％。除例9随访不足12个月，其他8例B-ALL移植后复发患者CAR-T治疗后1年OS率100.0％，4例患者无病生存2年以上，其中2例LFS期超过35个月，预期CAR-T细胞治疗后2年OS率为87.5％、2.5年OS率为52.5％。

6例移植后复发患者接受MSD来源CAR-T，治疗后中位OS、LFS时间分别为25.4、20.7个月，3例再次复发；3例采用Haplo来源CAR-T治疗的患者中，例2、例9无病生存35.7月、6.3月，1例（例7）再次复发（图2）。

**图2:**
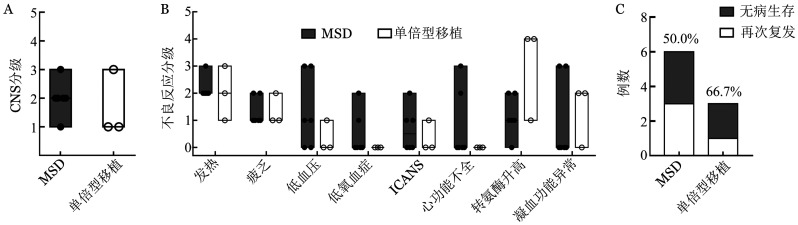
不同供者来源CAR-T细胞治疗B-ALL移植后复发患者的不良反应（A、B）和疗效（C） CAR-T细胞：嵌合抗原受体T细胞；B-ALL：急性B淋巴细胞白血病；CRS：细胞因子释放综合征；ICANS：免疫效应细胞相关的神经毒性；MSD：同胞全相合移植

四、供者CD19 CAR-T体内扩增和存留时相

CAR-T细胞回输后患者外周血CAR-T细胞占CD3^+^T细胞的比例（CAR-T/CD3^+^T ％）于回输后7～21 d达到峰值，CAR-T细胞数目达峰的中位时间为14 d。所有患者外周血CAR-T/CD3^+^T ％峰值的中位数为42％（14.7％～85.9％）（图3）。

**图3:**
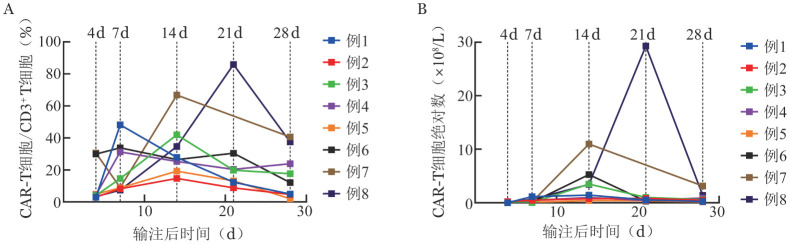
供者CD19 CAR-T细胞体内扩增和存留时相 CAR-T细胞：嵌合抗原受体T细胞

## 讨论

由于目前治疗手段有限、缓解率不高，成人ALL患者接受allo-HSCT后复发的治疗仍是难题。复发患者可接受化疗、DLI或者二次移植。DLI可增强移植物抗白血病（GVL）效应，DLI治疗复发ALL患者的中位OS期仅为9.8个月[1]，且易诱发GVHD等并发症[6]。二次移植后的患者1年和3年OS率仅有23％、11％[7]。

CAR-T细胞靶向特异性抗原杀灭体内肿瘤细胞，作为新型免疫疗法在复发难治性B-ALL取得了较其他方案更高的缓解率[2],[8]–[9]，供者CAR-T细胞则具有识别特异性抗原及同种异体抗原双重信号的优势。Kochenderfer等[10]首次报道CAR-T细胞用于治疗allo-HSCT后复发，后Chen等[11]–[12]、Brudno等[13]的研究也均显示CD19 CAR-T细胞对移植后复发ALL患者缓解率高、aGVHD发生率低。Chen等[12]的研究显示，85.7％（30/35）移植后复发ALL患者接受CD19 CAR-T细胞输注后获得CR，随访18个月的无复发生存（RFS）率、OS率分别为18.3％、30％。近期多个研究报道CD19 CAR-T治疗B-ALL移植后复发的缓解率（70～90％）明显高于传统治疗[12],[14]–[16]。本研究CAR-T细胞治疗28～42 d后评估，全部9例患者均达到骨髓MRD阴性的完全缓解和完全供者嵌合，例6的EMR也得到控制，中位随访25.1个月，6例患者获得无病生存，2例患者LFS期超过30个月，疗效与文献报道近似。

自体CAR-T细胞治疗难治复发B-ALL，若肿瘤细胞负荷低[2]、输注细胞剂量较高[8]或清淋巴细胞预处理强度较高[17]，缓解率较高，且自体CAR-T细胞输注前骨髓Blasts<5％且无EMR患者的OS期更长[2]。采用供者CD19 CAR-T治疗移植复发患者，目前未观察到肿瘤负荷、CAR-T输注量显著影响CR率[12],[14]，虽然Chen等[12]发现治疗前骨髓blasts≤10％的患者复发率较低（25％对78.6％，*P*＝0.006），但总体上再次复发率仍然较高，许多研究者报道随访1年复发率为40％～60％[12],[14]–[16]。本研究中均采用新采集供者细胞制备CAR-T细胞，且CD19 CAR均以4-1BB为共刺激信号，CR率达100.0％，有4例患者复发，中位复发时间为8.8个月，CAR-T治疗前复发状态均为骨髓MRD阳性，其中1例伴EMR（例6），虽CAR-T细胞治疗后数月内影像学检查无明显的髓外病情进展，但出现骨髓复发。因此，我们推测伴EMR是CAR-T细胞治疗后再复发的危险因素，但仅为个案现象，仍需要在更大样本研究中证实。

CAR-T细胞治疗后的长期疗效受限于再次复发，本研究中4例复发患者均是CD19阴性复发，且未采取抗GVHD预防，可能原因是CAR-T细胞在体内的存留时间短、CAR-T细胞耗竭继发肿瘤免疫逃逸。3例患者在化疗或二次移植后白血病进展。例4在CAR-T细胞治疗前经化疗联合DLI，疗效不理想，CAR-T后再次复发我们采用化疗诱导缓解后行二次移植，移植后缓解状态持续14个月，由于白血病恶性程度高，最终进展死亡。仅例8行CD22 CAR-T细胞治疗后无病生存，CD22 CAR-T可能是患者CD19 CAR-T细胞治疗再次复发后获得缓解的手段。为减少CAR-T后复发，可考虑联合细胞程序性死亡因子PD-1抑制剂[18]或DLI维持治疗[19]，也有学者尝试应用CD22-CD19双靶点CAR-T细胞等方法[20]–[22]。

CAR-T回输后患者的不良反应表现为轻到中度的CRS和（或）神经症状。例6不良反应较严重，出现3级CRS及多浆膜腔积液，可能是因其存在多发EMR灶而肿瘤负荷较高。严重CRS发生率低可能原因是60％患者CAR-T输注前肿瘤负荷均<5％。另有1例患者（例9）出现aGVHD。由于CAR-T免疫治疗细胞输注量低，aGVHD发生率低于DLI[23]，但GVHD的发生也可能与治疗前肿瘤负荷较高相关，Liu等[15]采用人源化CD19 CAR-T细胞治疗15例移植后复发患者，骨髓原始细胞中位数43.73％，10例患者发生aGVHD（Ⅰ～Ⅱ度6例、Ⅲ～Ⅳ度4例）。本组患者CAR-T细胞回输量（0.86～3.53）×10^6^/kg，且多数为MSD移植，单倍型移植患者仅3例，因此aGVHD发生率较低。

本研究尚存在一定局限性。其一，病例数少，且患者治疗前缓解深度不同；第二，移植方式不同，MSD移植比例为66.7％；第三，未通过脑脊液相关指标变化进一步了解发生CRES患者的情况。

本研究显示，供者CD19 CAR-T细胞治疗是B-ALL患者接受allo-HSCT后复发的可选治疗手段，疗效和安全性得到初步肯定，但对伴EMR患者疗效不佳、再复发风险高，由于样本量少，研究结果仍需大样本临床试验进一步验证。
